# Whitehead on Abortion and Sterility

**Published:** 1848-05

**Authors:** 


WHITEHEAD ON ABORTION AND STERILITY.

To the same house we are indebted for a copy of Mr. Whitehead's treatise on the *Causes and Treatment of Abortion and Sterility.* It is the result of an extended practical inquiry into the physical and morbid conditions of the uterus, with reference especially to leucorrhœal affections and the diseases of menstruation. Such are the advances made from year to year in this department of our profession, that the practitioner who does not conuslt the recent works on the complaints of females will soon find himself in the rear of his more studious brethren. This is one of the works which must be studied by those who would know what the present state of our knowledge is respecting the causes and treatment of abortion and sterility.

